# Selecting Adjuvant Treatment for Endometrial Carcinoma Using Molecular Risk Factors

**DOI:** 10.1007/s11912-019-0825-z

**Published:** 2019-07-31

**Authors:** Bastiaan G. Wortman, Remi A. Nout, Tjalling Bosse, Carien L. Creutzberg

**Affiliations:** 1 Department of Radiation Oncology, Leiden University Medical Center, K1-P, Albinusdreef 2, P.O. Box 9600, 2300 RC Leiden, The Netherlands; 2 Department of Pathology, Leiden University Medical Center, Leiden, The Netherlands

**Keywords:** Endometrial cancer, Adjuvant treatment, Radiotherapy, Chemotherapy, Molecular risk factors, Molecular alterations, Targeted therapies, Checkpoint inhibitors, PARP inhibitors

## Abstract

**Purpose of Review:**

To provide an overview of common molecular risk factors in endometrial cancer (EC) with the possibility to improve adjuvant treatment selection.

**Recent Findings:**

Recent studies have discovered and confirmed four different molecular subclasses in EC, with each having a distinct prognosis; *POLE-*ultramutated, microsatellite unstable, copy-number low, and copy-number high. Subsequent studies have shown that combining both molecular with clinicopathological risk factors can potentially improve adjuvant treatment selection for women with high-intermediate risk EC. For high risk and advanced stage EC, several molecular alterations are being explored for targeted therapy.

**Summary:**

Molecular alterations are frequently found in endometrial cancer and have currently not been implemented in the treatment guidelines for EC. Assessment of molecular alterations can distinguish patients that require less or more intensified adjuvant treatment. Trials investigating targeted therapies in EC are ongoing and have shown some promising results, however, more evidence is needed and results of randomized trials have to be awaited.

## Introduction

### Epidemiology

Endometrial cancer (EC) is the most common gynecological cancer in postmenopausal women in developed countries and the incidence is rising due to increased obesity and aging of the population. In the US, endometrial cancer is the 4th most common cancer in women with an estimated incidence of 61,880 new cases in 2019. Most women with EC are diagnosed between the ages of 60 and 80, and most are diagnosed at early stage of disease due to early symptoms such as vaginal bleeding. This generally results in a favorable prognosis and a relatively low number of cancer-related deaths (estimated 12,160 in 2019 in the US) [1, 2].

### Histology and Risk Factors

The most common histological type of EC is endometrioid adenocarcinoma (EEC), accounting for approximately 70–80% of EC. Non-endometrioid cancers (NEEC) mainly comprise serous and clear cell cancers, accounting for approximately 5–10% and 1–5% of EC, respectively, and the other aggressive subtypes are undifferentiated and dedifferentiated EC and uterine carcinosarcomas. Well-established clinicopathological risk factors, used in the current treatment guidelines, are age, histological type, tumor grade, International Federation of Gynecology and Obstetrics (FIGO)-stage, depth of myometrial invasion, and presence and extent of lymph-vascular space invasion (LVSI). EEC is graded using the FIGO grading system as low grade (grade 1), intermediate grade (grade 2), or high grade (grade 3) based on the proportion of solid growth and nuclear atypia, while the NEEC are high grade by definition. Using combinations of these risk factors, risk groups have been defined based on data from randomized trials, with each risk group having a distinct prognosis and adjuvant treatment recommendations have been determined for these risk groups (Table 1) [3].

**Table 1:** Risk groups in endometrial cancer as proposed by the ESMO-ESGO-ESTRO consensus guideline and related adjuvant treatment [3]

Risk group	Description	Current adjuvant treatments
Low	FIGO stage IA EEC: grade 1–2, LVSI neg.	NAT
Intermediate	FIGO stage IB EEC: grade 1–2, LVSI neg.	VBT *(NAT if age < 60)*
High-intermediate	FIGO stage IA/B EEC: grade 1–2, LVSI pos.	VBT (EBRT if stage IB LVSI pos.)
	FIGO stage IA EEC: grade 3, LVSI neg.	VBT *(NAT if age < 60)*
	FIGO stage IA EEC: grade 3, LVSI pos.	EBRT (VBT if LNI neg.)
High	FIGO stage IB EEC: grade 3	EBRT *(VBT if LVSI neg. or LNI neg.)*
	FIGO stage II EEC: grade 1–2, LVSI neg.	VBT
	FIGO stage II EEC: grade 1–2, LVSI pos.	EBRT *+/− VBT boost*
	FIGO stage II EEC: grade 3 or LVSI pos.	EBRT
	FIGO stage III EEC	EBRT + CT*
	FIGO stage IA SC/CC, LVSI neg.	*VBT*
	FIGO stage > IB SC/CC	*EBRT + CT*

*FIGO*, International Federation of Gynecology and Obstetrics 2009; *EEC*, endometrioid endometrial cancer; *LVSI*, lymph-vascular space invasion (neg.: negative, pos.: substantial LVSI); *NAT*, no adjuvant treatment; *VBT*, vaginal brachytherapy; *LNI*, lymph node involvement (surgical staged); *EBRT*, external beam radiotherapy; *CT*, chemotherapy; *SC*, serous carcinoma; *CC*, clear cell carcinoma

*Italic*: to be considered

*EBRT and chemotherapy either combined (PORTEC-3 and GOG 258 schedule) or sequentially

### Treatment

Women with EC are primarily treated with surgery, consisting of abdominal or laparoscopic hysterectomy and bilateral salpingo-oophorectomy, with or without lymph node evaluation. The indication for adjuvant treatment has been based on the presence of clinicopathological risk factors. Women with low or low-intermediate risk EC are treated with surgery alone [4, 5]. Women with high-intermediate risk (HIR) EC usually receive adjuvant radiotherapy, mainly vaginal brachytherapy (VBT) [6–8]. Women with high-risk EC (HR), being at higher risk of recurrence, receive pelvic external beam radiotherapy (EBRT) with or without adjuvant chemotherapy. Especially in the case of substantial LVSI, EBRT is preferred over VBT alone to maximize pelvic nodal control in HIR EC [9–11]. The role of adjuvant chemotherapy, most often given in combination with EBRT, has been the subject of recent randomized trials, which showed increased relapse-free survival rates with combined adjuvant treatment at the cost of increased toxicity, and this is mainly recommended in stage III disease and for serous cancers [12, 13].

### Molecular-Genetic Characterization of EC and Molecular Risk Factors

**Fig. 1:**
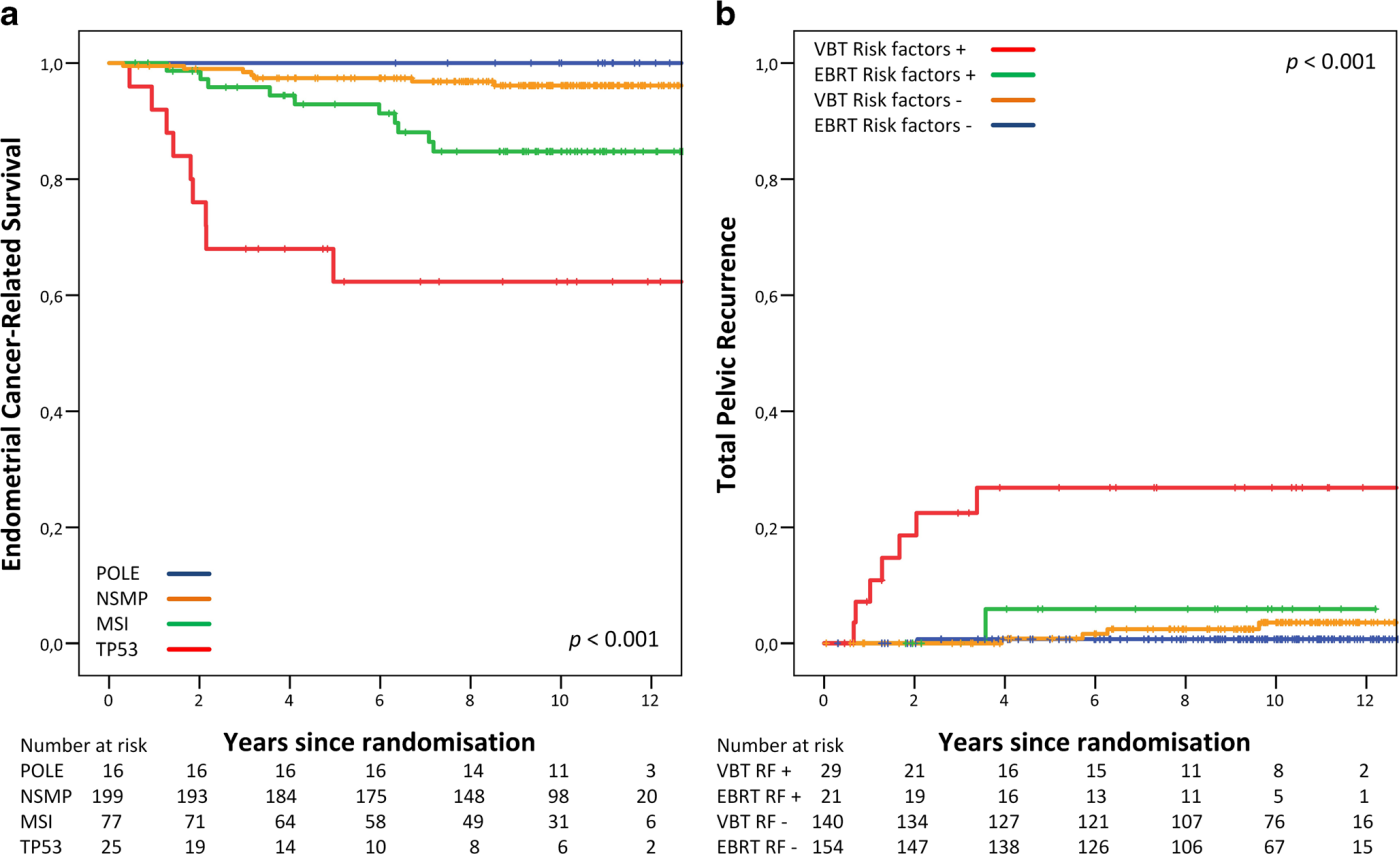
**a** Endometrial cancer-related survival by 4 molecular subgroups in the PORTEC-2 trial for high-intermediate risk EC. **b** Total pelvic recurrence by unfavorable risk factors (LVSI, p53-mutant, or L1CAM expression). Reproduced from Wortman, B.G., et al., *Ten-year results of the PORTEC-2 trial for high-intermediate risk endometrial carcinoma: improving patient selection for adjuvant therapy.* Br J Cancer, 2018. **119**(9): p. 1067-1074, which is distributed under the terms of the Creative Commons Attribution 4.0 International License (http://creativecommons.org/licenses/by/4.0/), courtesy of the authors

The extensive molecular-genetic characterization of endometrial cancer by the Cancer Genome Atlas Group (TCGA) has been pivotal in understanding the molecular pathways involved in endometrial cancer development and their prognostic implications. By full genomic analysis of 373 EC cases, 4 different molecular subclasses were identified based on mutation rates and somatic copy-number alterations (SCNA). The ultramutated subclass, characterized by mutations in the exonuclease domain of DNA polymerase-epsilon (*POLE)*, is associated with a very favorable prognosis. The hypermutated subclass based on microsatellite instability (MSI) has been shown to have an intermediate prognosis. The copy-number low subclass with low mutation frequency (also called subclass with no specific molecular profile or NSMP) has also been associated with an intermediate prognosis. The copy-number high subclass, characterized by *TP53* mutations, with mainly serous-type EC, has a very high degree of SCNAs and a low mutation rate and is associated with the most unfavorable prognosis [14••]. Several research groups have reproduced and validated the four TCGA subclasses in formalin-fixed, paraffin-embedded tissues in different EC cohorts by using their surrogate markers (Fig. 1a). These findings have led to a clinically available molecular classification tool for diagnosis and decision making [11•, 15, 16•, 17•, 18, 19•].

The current guidelines for diagnosis and treatment of EC are based on the clinicopathologic factors, age, FIGO stage, histologic type and grade, myometrial invasion, and the presence of LVSI, but do not include molecular alterations. The current question is if and how these molecular risk factors can be used to guide adjuvant treatment. The purpose of this review was to provide an overview of the molecular risk factors involved in endometrial cancer in relation to their prognostic implications and potential therapeutic consequences.

## Methods

### Data Sources and Study Selection

A PubMed search was performed including articles published between 1 January 2000 and 18 December 2018. Search terms included: endometrial cancer AND adjuvant radiotherapy OR adjuvant chemotherapy OR adjuvant chemoradiotherapy AND molecular pathways OR molecular risk factors. Preclinical research involving animal studies or endometrial cancer cell lines were excluded, as well as articles on uterine sarcomas. This search strategy resulted in 383 articles, which were subsequently selected on title, abstract, and full text. Additionally, review of the reference lists of these articles was performed and relevant papers were included.

## Results

### Molecular Characterization

Over the last few years, more specific knowledge of molecular alterations and molecular heterogeneity within EC has become available [14••]. The discoveries reported by TCGA have led to a great number of studies involving molecular characteristics and novel targets for therapy of EC (Table 2).

**Table 2:** Common molecular alterations in endometrial cancer.

Molecular (pathway) alteration	Frequency in EC	Description	Prognosis	Potential targeted therapies
*POLE* mutation	6–12%	DNA repair	Excellent	PD-1/PD-L1 immune checkpoint inhibitors*
MMRd	20–40%	DNA repair	Intermediate	PD-1/PD-L1 immune checkpoint inhibitors*
*TP53* mutation	9–29%	Tumor suppressor	Poor	PARPi; platinum derivatives
L1CAM overexpression	16–28%	Cell adhesion/signaling protein	Poor	n/a*
ER/PR expression	72–95%	Hormone receptors	Good	Endocrine therapy (with PI3K/mTOR inhibitors)
Wnt-ß-catenin pathway	18–25%	Wnt signaling pathway	Intermediate	n/a
PI3K-AKT-mTOR pathway	> 80%	PI3K/AKT/mTOR signaling pathway	Good-intermediate	PI3K, AKT, mTOR inhibitors
HER2/Neu overexpression	14–47%	Epidermal growth factor receptor	Poor	Monoclonal antibodies, protein kinase inhibitors
*ARID1A* mutation	30–40%	Tumor suppressor	Good-intermediate	EZH2 inhibitors

*EC*, endometrial cancer; *n/a*, not available. For abbreviations see text

*The addition of PARP inhibitors (PARPi) has been explored for several molecular alterations, however evidence of efficacy is still limited

#### *POLE*-Mutant Subclass

Approximately 6 to 12% [14••, 16•, 17•, 18, 19•, 20, 21•] of all EC carries a pathogenic variant in the exonuclease domain of DNA polymerase epsilon (POLE) and are more frequently found in relatively younger women (median age 58–63.5 vs. 66–68.5) with lower BMI and higher grade endometrioid endometrial tumors compared to *POLE* wildtype EC [21•, 22, 23]. It is thought that *POLE* mutations impair the proofreading function during DNA replication that leads to an exceptionally high mutational burden. In *POLE-*mutant EC, an increased antitumor response by peritumoral and tumor-infiltrating CD8+ lymphocytes has been reported, most probably because the mutated DNA fragments act as neo-antigens that elicit a strong immune response [24–28]. In contrast to the aggressive appearance and high grade of *POLE*-mutant EC, they have consistently been shown to have an excellent prognosis with only an occasional relapse, both with and without adjuvant treatment. It has been suggested that their very favorable outcome is mainly based on the strong host immune response and that adjuvant (radio) therapy in early stage *POLE*-mutant EC could be safely omitted [16•, 17•, 18, 19•, 21•, 22, 23, 29].

#### MSI Subclass

The TCGA has defined a microsatellite unstable (MSI) subclass; however, in clinical practice, mismatch repair deficiency (MMRd) is tested by immunohistochemistry of the MMR proteins. This subclass comprises between 20 and 40% of all EC [14••, 16•, 17•, 30, 31•, 32]. MMRd EC fails to express one or more of the MMR proteins MLH1, MSH2, MSH6, or PMS2, leading to the accumulation of mismatches, deletions, and microsatellite instability. The majority (~ 95%) of MMRd EC is MMR deficient due to *MLH-1* promoter hypermethylation with subsequent loss of MLH1 protein expression. The remaining 3–6% consists of MMRd EC cases due to either biallelic somatic mutations or germline defects in one of the MMR genes (Lynch syndrome) [33]. MMRd EC shows a similar increase in tumor-infiltrating lymphocytes as seen in *POLE-*mutant EC, however, it is associated with negative prognostic factors such as higher histologic grade, presence of LVSI and with older age, and advanced stage (III/IV). MMRd EC has an intermediate prognosis in most studies [30, 31•, 32, 34–36].

#### Copy-number High Subclass

In this subclass characterized by a high number of somatic copy-number alterations and a relatively low mutation rate, 90% of all tumors harbor somatic *TP53* mutations. This subclass consists of serous and serous-like subtypes and is associated with unfavorable overall and progression-free survival. Both amplification of the human epidermal growth factor receptor 2 (Her-2/Neu) and homologous recombination deficiency (HRd) are frequent molecular alterations in this subclass [14••, 17•, 37•].

#### Copy-number Low Subclass

Within the copy-number low (CNL) subclass, no specific molecular driver could be identified. This large subclass is characterized by a low mutational burden, comprises most endometrioid-type cancers of low to intermediate grade and is associated with an intermediate prognosis. Frequent molecular alterations in this subclass are *CTNNB1* mutations (~ 52%) and PI3K pathway alterations [14••]. The CNL subclass contains a heterogeneous set of tumors, which complicates the distinction of favorable or unfavorable tumors. A subgroup with amplification of 1q32.1 has been reported to be associated with impaired cancer-specific and recurrence-free survival [38]. On the contrary, among all grade 3 ECs, the CNL subclass is associated with a favorable prognosis as compared with the other grade 3 ECs [39•].

### Additional Molecular Characteristics and Pathways

#### Endocrine Receptors

Expression of estrogen (ER) and progestogen (PR) receptors is common in EC, especially in EEC, with a reported frequency of 72–81% [40]. ER and PR can stimulate or inhibit the transcription of several genes and are associated with low-grade tumors, more favorable tumor histology, and favorable prognosis. Loss of ER or PR expression is related to higher grade tumors and impaired disease-free survival [41, 42]. Targeting the endocrine receptors by hormonal therapy is used for women with low-grade early stage EC that wish to preserve fertility and also for those with low-grade metastatic EC [3]. Hormonal therapy in women with grade 1 metastatic EEC, especially those with lung or oligometastasis, can provide durable responses [43].

#### PI3K-AKT-mTOR Pathway

The PI3K-AKT-mTOR pathway is essential for regulating diverse cellular responses including cell growth and survival and is altered in over 80% of all ECs [14••, 44, 45]. Somatic loss of *PTEN*, a tumor suppressor gene, is the most frequent alteration in this pathway. Other common alterations are mutations in the *PIK3CA*, *PIK3R1*, and *KRAS* genes. While loss of *PTEN* is often correlated with endometrioid histology, low tumor grade, and favorable prognosis, *PIK3CA* is associated with high tumor grade. As PI3K-AKT-mTOR is the most common pathway alteration in EC, many therapeutic options have been explored. Targeted therapy of the PI3K pathway by using PI3K inhibitors as single agents has not shown satisfactory results, and novel dual PI3K-mTOR, mTOR, and AKT inhibitors are being investigated. In previous studies, it has been suggested that PI3K-AKT-mTOR pathway inhibition suppresses resistance to endocrine therapy in several solid tumors, including EC [45–48]. Promising results have been described for combined treatment with PI3K inhibition and endocrine therapy, and the combination of everolimus and letrozole has been shown to lead to 53% response rates in chemo-naïve patients with recurrent EC [49, 50•].

Metformin has also been linked to regulation of PI3K-AKT-mTOR signaling and activates the AMP protein kinase (AMPK) pathway, which by several processes, regulates cell growth via suppression of mTOR. The clinical activity of metformin has been investigated; however, a recent randomized placebo-controlled phase 3 window trial in women with endometrial hyperplasia and EEC did not show reduction of tumor proliferation [46, 51].

#### ARID1A

Mutation of the AT-rich interactive domain 1A (*ARID1A*) gene has been found in approximately 30–40% of EEC and only in 0–7% of serous EC [14••, 52, 53]. ARID1A is involved in the switch/sucrose non-fermentable (SWI/SNF) chromatin remodeling complex and encodes a nuclear protein, BAF250a, that is involved in essential cell-cycle control and DNA damage repair pathways. Loss of BAF250a protein expression can result in cell proliferation and malignant progression of precursor lesions. A possibility for targeted therapy in *ARID1A*-mutated EC by inhibition of its downregulator EZH2, a methyltransferase enzyme, has been described, however, no (randomized) clinical trials have been performed so far [52–55]. Other suggestions for targeted therapy in this particular subgroup are PI3K inhibitors, as previous studies have described a crosstalk between ARID1A and the PI3K-AKT-mTOR pathway [54].

#### ERBB2 Receptors

The epithelial growth factor (EGF) system consists of four receptors EGFR, HER-2/Neu, HER-3, and HER-4. HER-2/Neu amplification and/or overexpression are most frequently found in serous EC, with wide variations in reported frequencies of amplification and overexpression, which range between 14–80% and 21–47%, respectively [56]. This can be explained by different testing or scoring methods used and test result interpretation, as currently, no standardized testing methods for HER-2/Neu in EC exist. In order to successfully perform clinical trials of HER-2/Neu-targeted therapy, identification of patients that might benefit the most from this therapy is a key aspect of eligibility. Previous studies have suggested that the HER-2/Neu receptor might be a potential target for therapy, however, studies using HER-2/Neu inhibitors as monotherapy have had disappointing results [56–59]. In a recent study, the combination of trastuzumab with carboplatin and paclitaxel chemotherapy in HER-2/Neu-positive serous carcinomas resulted in a prolonged median progression-free survival of 13 months compared to 8 months in the carboplatin-paclitaxel alone group (*p* = 0.005) [60•].

#### Wnt-ß-catenin Pathway

Molecular alterations in the Wnt-ß-catenin pathway are found in approximately 52% of EC within the NSMP subclass and approximately 18–25% of ECs in general [14••, 44, 61, 62]. ß-catenin acts as a transcriptional factor in the Wnt-pathway that regulates gene transcription and development. Estrogens can induce Wnt/ß-catenin signaling, which may result in growth stimulation of endometrial hyperplasia and well-differentiated EC, while progesterone can inhibit this process [63]. In the normal situation, ß-catenin levels are kept low through degradation by the ubiquitin-proteasome pathway [64]. In ß-catenin (*CTNNB1)-*mutated EC, the Wnt signaling pathway is activated by nuclear accumulation of ß-catenin that results in EC progression and abnormal expression of cell proliferation and progression genes. *CTNNB1* exon 3 mutation is associated with decreased overall survival [62, 65].

#### Programmed Cell Death-1/Ligand-1

Both *POLE-*mutant and MMRd ECs contain a high mutational burden which elicits an increased immune response by tumor infiltrating lymphocytes (TIL). It has been shown that hypermutated tumors harbor higher neoantigen loads, which are associated with increased immune response by cytotoxic CD8+ TILs. In a response to this, the tumor upregulates immune-inhibitory molecules like PD-1/PD-L1 and others [25, 26]. Increased PD-1 and PD-L1 expression have made these tumors attractive for immune checkpoint inhibitors acting against PD-1 receptor or its ligand PD-L1 [25, 28•, 29, 36, 66, 67•]. Previous trials have confirmed this hypothesis and have shown remarkable clinical responses to nivolumab, pembrolizumab, and lenvatinib in women with recurrent or metastatic hypermutated tumors, including EC [68–70]. Pembrolizumab has been approved by the FDA for the treatment of women with metastatic MMRd EC.

#### Homologous Recombination Deficiency

A relative frequent DNA repair defect, HRd, has recently been observed especially in the *TP53*-mutated or serous-like subclass. Homologous recombination is essential for repair of DNA double-strand breaks, which is mediated by (among others) BRCA1 and BRCA2 proteins. In a study of 24 EC, HRd was analyzed by detecting RAD51 foci after irradiation of tissue samples, and HRd was found in 24% of EC. All 6 HRd EC were non-endometrioid cancers, especially serous type EC, and in 67% of all NEEC, HRd was found. In 5 of the 6 HRd EC, a molecular basis for HRd could be identified by somatic copy-number alterations in the BRCA core or related genes. Targeting HRd in EC by using platinum-based chemotherapy and/or PARP inhibitors seem promising therapeutic options [37•].

PARP, or poly (ADP-ribose) polymerase is involved in DNA damage detection and generation of poly (ADP-ribose) chains. These chains facilitate chromatin remodeling and DNA repair. Loss of PARP results in persistent single-strand DNA breaks and eventually in double-strand DNA breaks. Normally, DSBs are repaired by homologous recombination or other repair mechanisms such as non-homologous end joining. Tumor cells with either loss of PARP or with HRd alone are still viable, however, in case of simultaneous inhibition of both repair pathways, accumulation of DSBs leads to cell death [71•, 72]. Objective responses to PARP inhibitors (PARPi) have been reported in women with HRd (BRCA mutant or BRCA wildtype) ovarian carcinoma and in BRCA-mutated breast cancer [73, 74]. Another useful consequence of PARPi is the increase of neo-antigen expression caused by the accumulating DNA damage, resulting in an increased anti-tumor immune response, suggesting that a combination of PARPi with immune checkpoint inhibitors might be an effective and even synergistic treatment. Most promising would be the use of this combination in the subgroup of patients with hypermutated ECs with high TIL counts (*POLE*-mutant or MMRd EC) and those with HRd EC [37•, 71•, 72].

## Limitations of the Current Guidelines

The molecular discoveries by the TCGA have provided new insights into the carcinogenesis and clinical behavior of endometrial cancer. EC within the subclass with *POLE* mutation have shown an excellent prognosis, even without further adjuvant therapy, while EC with unfavorable molecular alterations have shown an increased risk of locoregional recurrence and distant spread of the disease. These molecular risk factors have not yet been included in clinical guidelines [3]. Combining molecular features and clinicopathological risks factors into a stratification model holds promise to guide surgery, adjuvant treatment, and disease surveillance, which results in more individualized adjuvant treatment and reduction of both under and overtreatment [16•, 17•]. Furthermore, a combined assessment can lead to a more objective approach of EC classification, compared to the current histology-based classification, which has its limitations as inter-observer variability [75, 76]. Apart from this, other strategies such as the molecular assessment of endometrial biopsies, which has shown high concordance with the final hysterectomy specimen, could guide both (pre-)surgical treatment decisions such as the extent of surgical staging [77, 78].

## Integrated Molecular Risk Profile and Adjuvant Treatment

In a comprehensive translational research project, 834 HIR EC samples of the pooled PORTEC-1 and 2 biobank were analyzed, and the TCGA subclasses and other molecular risk factors such as *CTNNB1* exon 3 mutations were combined with clinicopathologic factors such as presence of LVSI and with L1-cell adhesion molecule (L1CAM) overexpression [9, 10, 17•]. L1CAM is a membrane glycoprotein with an important role in tumor cell adhesion and migration. L1CAM overexpression (> 10%) on immunohistochemistry is reported in approximately 16–28% of EC and is associated with frequent *TP53* mutation, non-endometrioid histology, histological grade 3, LVSI, and with an increased risk of locoregional and distant spread. It is independently related to decreased overall and relapse-free survival [79–82, 83•, 84].

Combining all these risk factors resulted in 3 subgroups within HIR EC with favorable, intermediate and unfavorable outcomes. The favorable subgroup with either *POLE*-mutant EC or with absence of other risk factors had an excellent prognosis, for which it was suggested that adjuvant treatment could safely be omitted; the unfavorable subgroup, strongly associated with the risk of locoregional and distant spread, with either *TP53*-mutation, L1CAM overexpression, or substantial LVSI, for which more intensive adjuvant treatment by EBRT seems justified (Fig. 1b), and an intermediate subgroup with either MMRd or *CTNNB1* mutation [11•]. For this intermediate group, most benefit of the standard treatment VBT is expected. This molecular-integrated risk model with corresponding consequences for adjuvant treatment is currently being investigated in the international, multicenter randomized controlled PORTEC-4a trial, of which recent evaluation of the pilot phase showed feasible trial logistics and a satisfactory patient acceptance rate [85, 86].

## Conclusions and Future Directions

Molecular alterations are frequently found in endometrial cancer and increasing knowledge on their prognostic significance and possible therapeutic options has been gained. Trials investigating (adjuvant) treatment based on molecular alterations are ongoing for women with high-intermediate risk, high risk, and recurrent or metastatic EC. More evidence on selecting adjuvant radiotherapy, chemotherapy, and targeted therapies based on molecular alterations in EC are needed and results of ongoing and future trials investigating adjuvant and targeted therapies for EC are eagerly awaited.
